# The Effect of PtRuIr Nanoparticle Crystallinity in Electrocatalytic Methanol Oxidation

**DOI:** 10.3390/ma6051621

**Published:** 2013-04-29

**Authors:** Yanjiao Ma, Rongfang Wang, Hui Wang, Shijun Liao, Julian Key, Vladimir Linkov, Shan Ji

**Affiliations:** 1 Key Laboratory of Eco-Environment-Related Polymer Materials of Ministry of Education of China, College of Chemistry and Chemical Engineering, Northwest Normal University, Lanzhou 730070, China; E-Mails: myjmm@126.com (Y.M.); wanghui3931@126.com (H.W.); 2 Key Laboratory of Fuel Cell Technology of Guangdong Province, South China University of Technology, Guangdong, Guangzhou 510640, China; E-Mail: chsjliao@scut.edu.cn; 3 South African Institute for Advanced Materials Chemistry, University of the Western Cape, Cape Town 7535, South Africa; E-Mails: joolskey@yahoo.com (J.K.); vlinkov@uwc.ac.za (V.L.)

**Keywords:** electrocatalysts, fuel cells, methanol oxidation, structure, crystallinity

## Abstract

Two structural forms of a ternary alloy PtRuIr/C catalyst, one amorphous and one highly crystalline, were synthesized and compared to determine the effect of their respective structures on their activity and stability as anodic catalysts in methanol oxidation. Characterization techniques included TEM, XRD, and EDX. Electrochemical analysis using a glassy carbon disk electrode for cyclic voltammogram and chronoamperometry were tested in a solution of 0.5 mol L^−1^ CH_3_OH and 0.5 mol L^−1^ H_2_SO_4_. Amorphous PtRuIr/C catalyst was found to have a larger electrochemical surface area, while the crystalline PtRuIr/C catalyst had both a higher activity in methanol oxidation and increased CO poisoning rate. Crystallinity of the active alloy nanoparticles has a big impact on both methanol oxidation activity and in the CO poisoning rate.

## 1. Introduction

Increasing the electrocatalytic activity and stability of Pt-based catalysts has been the focus of much recent research [1,2,3] and remains a critical requirement for the future implementation of direct methanol fuel cells (DMFCs). Among the various Pt-based binary catalysts, the PtRu alloy has been reported as the most effective for methanol electro-oxidation [4,5,6], with further recent gains in activity and durability reported by incorporating a third metal, such as Co, Ni, Sn, Ir, *etc.* [7,8,9,10]. Among these ternary alloy catalysts, the PtRuIr/C system seems particularly promising [11,12,13]. Furthermore, the effect of composition for PtRuIr/C catalyst was systematically studied. However, the effect of its structure and morphology on methanol electro-oxidation is not focused on by other researchers.

Synthesis of nanostructured electrocatalysts is of great importance in developing the so-called “next-generation” catalysts [14]. The catalytic activity of such nanostructured electrocatalysts is highly dependent on the surface area, surface atomic structure, crystal size and shape. With control of nanostructure and morphology, large surface areas and abundant catalytic active sites can be realized, which enhance catalytic performance and utilization efficiency of the electrocatalyst [15]. In particular, amorphous structures in alloys can present unique compositions and catalytic surface structures as compared to conventional crystallized metal [16,17]. Some studies show that amorphous composition can have positive effects on the kinetics or stability of the methanol oxidation reaction due to amorphous alloys presenting unique compositions and surface structures for molecular reactions [18], while others show that intermetallic compounds with high-crystallinity have higher electrocatalytic activity for methanol oxidation reaction [19,20].

Inspired by the reports, the present work aimed to gain deeper insight into the effect of PtRuIr nanoparticle crystallinity on methanol electro-oxidation for carbon-supported PtRuIr catalysts. To this end, crystalline and amorphous carbon-supported PtRuIr structures were prepared, and then studied and compared using cyclic voltammetry and chronoamperometry.

## 2. Results and Discussion

**Figure 1:**
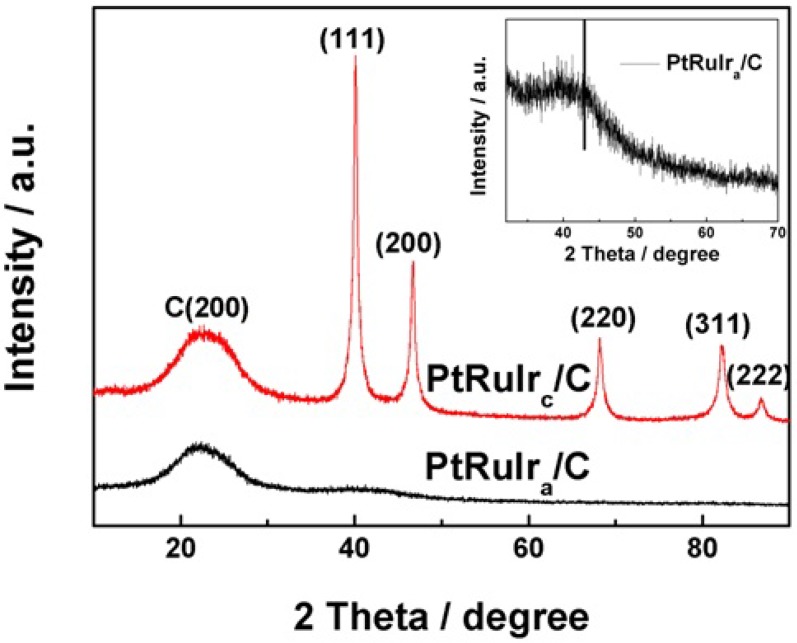
X-Ray Diffraction (XRD) patterns of PtRuIr_c_/C and PtRuIr_a_/C catalysts.

X-Ray Diffraction (XRD) analysis (Figure 1) produced clear differences in the peak distributions of the carbon-supported PtRuIr_c_/C (crystalline form) and PtRuIr_a_/C (amorphous form) catalysts. In the diffractograms of the two catalysts, the first peak located at about 24.8° in all the XRD plots is associated with the Vulcan XC-72R support, and no peaks corresponding to the metals Ir and Ru were observed [11]. For clarity, the diffraction patterns of the PtRuIr_a_/C catalyst between 32° and 70° have been enlarged in the inset of Figure 1. Here, the PtRuIr_a_/C catalyst had only one wide, diffuse, broad peak at approximately 2*θ* = 45°, indicating that the sample’s internal structure was amorphous [18]. In contrast, the XRD pattern of the heat treated sample, PtRuIr_c_/C, have the five main characteristic peaks of the face-centered cubic (fcc) crystalline Pt alloy [13,21,22], corresponding to the planes (111), (200), (220), (311), and (222), at 2*θ* values of *ca*. 40°, 47°, 68°, 82°and 86°, respectively. On the other hand, a displacement of the peaks related to the polycrystalline Pt towards more positive values of 2*θ* is observed. This can be ascribed to the existence of alloys between the metals Pt, Ru and Ir. The formation of alloy results in a contraction of the crystalline lattice of Pt due to the substitution of some atoms of Pt with large size (*r*_Pt_ = 0.138 nm) for the atoms of Ir and/or Ru with small sizes (*r*_Ru_ = 0.134 nm) (*r*_Ir_ = 0.136 nm) [11,23]. These results indicate that the PtRuIr_c_ alloy supported on carbon catalyst had fcc crystalline structure.

**Figure 2:**
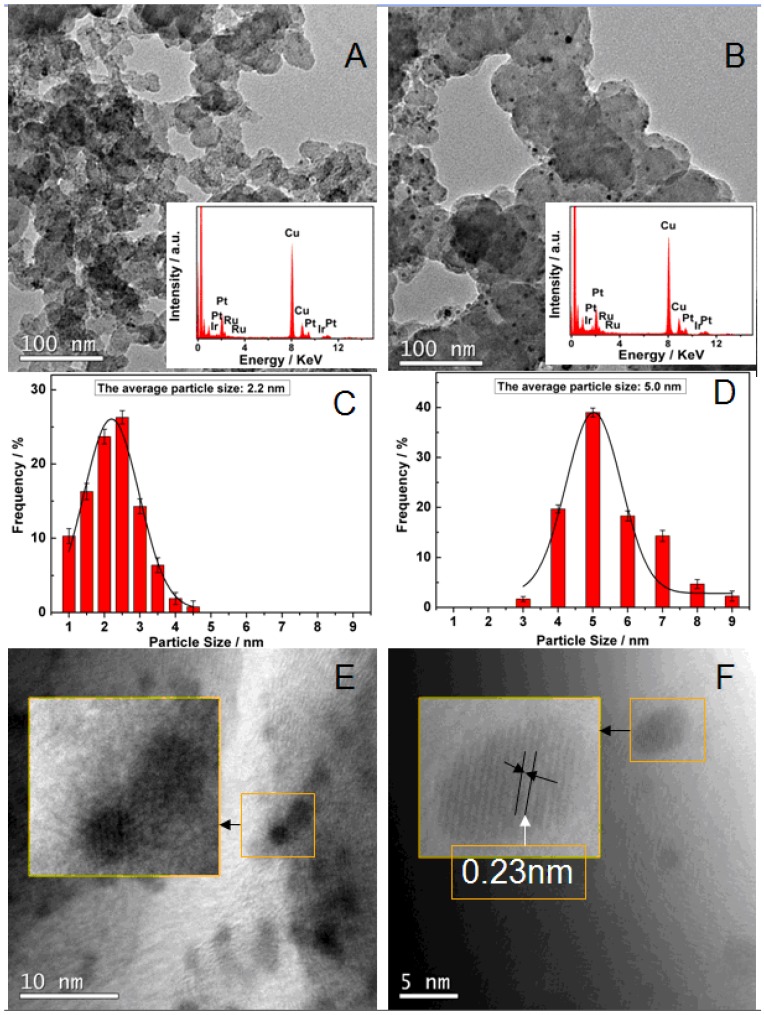
TEM, the corresponding particle size distributing histogram and HRTEM images of PtRuIr_a_/C (**A**,**C**,**E**) and PtRuIr_c_/C (**B**,**D**,**F**) catalysts. Inset of (**A**) and (**B**): EDX spectrum of the PtRuIr_a_/C (**A**) and PtRuIr_c_/C (**B**) catalysts.

Figure 2 shows TEM images, corresponding particle sizes distribution histogram and EDX composition of PtRuIr_a_/C and PtRuIr_c_/C. From the Figure 2A (PtRuIr_a_/C) and 2B (PtRuIr_c_/C), it can be observed that both catalysts were highly dispersed on the carbon support. The particle size distribution histogram of PtRuIr_a_/C (Figure 2C) and PtRuIr_c_/C (Figure 2D) catalyst based on examination of more than 300 particles show that the particle size varied from 1.0 to 4.5 nm for PtRuIr_a_/C and 3 to 9 nm for PtRuIr_c_/C and a relatively narrow size distribution for both catalysts. The derived average particle size is about 2.2 ± 0.02 nm and 5.0 ± 0.02 nm for the PtRuIr_a_/C and PtRuIr_c_/C catalysts (see Table 1), respectively. The HRTEM image of PtRuIr_a_/C in Figure 2E shows an inexplicit lattice, indicating that the particles of PtRuIr_a_/C are of mainly amorphous state [24]. In contrast, the HRTEM image (insets in Figure 2F) reveals that the PtRuIr_c_/C nanoparticles are crystalline, showing a lattice of ~0.23 nm identifiable as the d-spacing of the (111) plane of face-centered cubic Pt [25]. The EDS results of PtRuIr_c_/C and PtRuIr_a_/C (insets in Figure 2A,B) indicate that the both catalysts consist of: C, Pt, Ru and Ir, and *ca*. 3.5:3:1 of atom ratio for Pt:Ru:Ir is obtained. The result is also confirmed by ICP analysis. The metal loading for the two catalysts is *ca.* 20%, close to the normal value.

**Figure 3:**
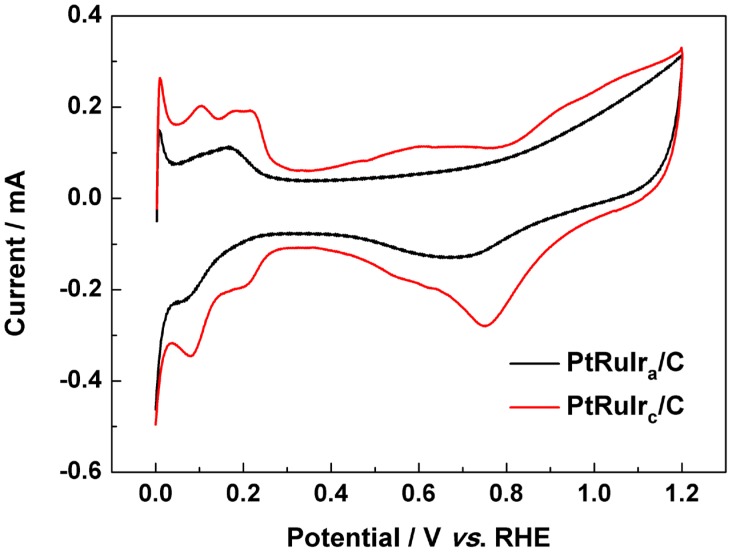
Cyclic voltammograms of PtRuIr_a_/C and PtRuIr_c_/C catalysts in 0.5 mol L^−1^ H_2_SO_4_ solution under N_2_ atmosphere; scan rate = 50 mV s^−1^.

Typical cyclic voltammograms (CVs) of PtRuIr_c_/C and PtRuIr_a_/C catalysts in 0.5 mol L^−1^ H_2_SO_4_ solution are shown in Figure 3. A well-defined CV feature of polycrystalline Pt is observable in the curve generated from PtRuIr_c_/C. Here, there are three pairs of redox peaks around 0.09, 0.173 and 0.214 V (*vs.* RHE), corresponding to the planes (110), (111), and (100), which can be ascribed to hydrogen adsorption/desorption on crystal surface sites of Pt [7,26]. In contrast, the CV curve of PtRuIr_a_/C catalyst only has one large, broad peak and does not exhibit the typical peaks of pure polycrystalline Pt between 0 and 0.3 V (*vs*. RHE). This further suggests that the active components of PtRuIr_a_/C catalyst had an amorphous structure. Furthermore, the oxide (OH_ads_) stripping peak (0.75 V *vs.* RHE) of the PtRuIr_c_/C is 70 mV more positive than that of PtRuIr_a_/C (0.68 V *vs.* RHE), suggesting faster hydroxyl desorption from the PtRuIr_c_/C surfaces [27].

**Table 1:** Composition the average particle size, and the electrochemical performance of the PtRuIr_a_/C and PtRuIr_c_/C catalysts.

Catalyst	PtRuIr_a_/C	PtRuIr_c_/C
Pt:Ru:Ir atom ratio	3.5:3.0:1.0	3.5:3.0:1.0
The average particle size/nm	2.2 ± 0.02	5.0 ± 0.02
*ECSA*/m^2^ g^−1^_metal_	59.5	32.6
The onset potential for CO oxidation/mV *vs.* RHE	663	521
The onset potential for methanol oxidation/mV *vs.* RHE	370	338
The mass activity for methanol oxidation/mA mg^−1^	147	298
The specific activity for methanol oxidation/mA cm^−2^	0.25	0.91

**Figure 4:**
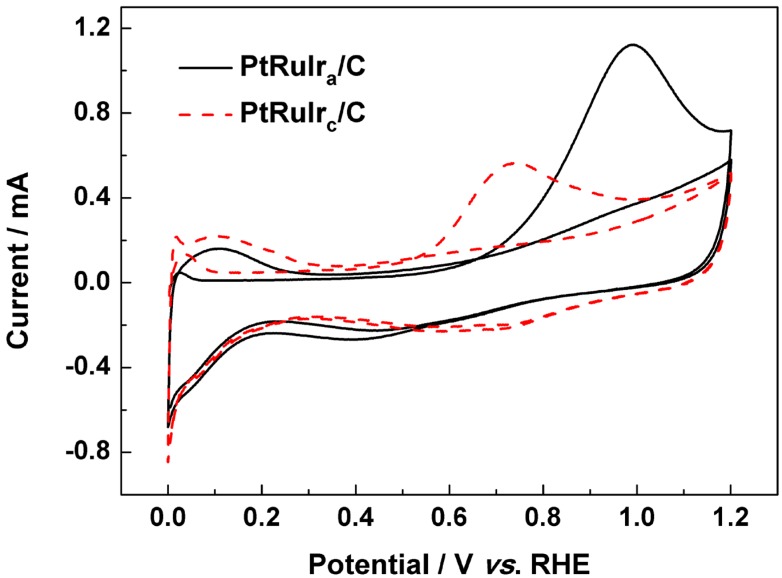
CO stripping voltammograms of PtRuIr_c_/C and PtRuIr_a_/C catalysts in a solution of 0.5 mol L^−1^ H_2_SO_4_ at room temperature.

The CVs for CO electro-oxidation on PtRuIr_c_/C and PtRuIr_a_/C catalysts are shown in Figure 4. Here, the hydrogen desorption peaks were completely suppressed in the first scan in the lower potential region (0 to 0.3 *vs.* RHE), due to the saturated coverage of CO_ads_ species on the surface of PtRuIr alloy active sites [28]. However, hydrogen desorption peaks recovered in the second cycle after the CO was removed by oxidation.

It can be seen from Table 1 that the onset potential of CO electro-oxidation with PtRuIr_c_/C (0.54 V *vs.* RHE) is lower than that of PtRuIr_a_/C (0.67 V *vs.* RHE), which demonstrates that crystallinity of PtRuIr alloy influences the CO oxidation ability (the onset oxidation potential). The peak potential on the PtRuIr_c_/C catalyst (0.73 V *vs.* RHE) show a negative shift of around 0.45 V (*vs.* RHE) compared to the PtRuIr_a_/C catalyst (0.98 V *vs.* RHE). The lower peak potential and onset potential of the CO_ads_ oxidation on PtRuIr_a_/C indicate that PtRuIr_c_/C catalyst was kinetically more active for CO_ads_ oxidation [17]. The electrochemical surface area (*ECSA*) of the catalyst was calculated using the Equation (1) [29]:

(1) $${ECSA}_{CO}=\frac{Q_{CO}}{484\omega }$$

where *Q*_CO_ is the charge for CO desorption electro-oxidation in microcoulomb (μC), 484 is the charge required to oxidize a monolayer of CO on the catalyst in μC cm^−2^ and *ω* is the precious metal loading, respectively. The *ECSA*_CO_ for PtRuIr_c_/C and PtRuIr_a_/C were 32.6 m^2^ g^−1^_metal_ and 59.5 m^2^ g^−1^_metal_ respectively. PtRuIr_c_/C had a lower *ECSA*_CO_ than the PtRuIr_a_/C.The real electrochemical surface area is determined by the active sites on the surface of the metal particle. The number of the active sites is related to the composition of the surface, the size of the particle and the structure of the surface [30]. After heat treatment, the PtRuIr_c_/C nanoparticles agglomerated resulting in increased particle size, which has been proved by TEM. The large particle size results in the small *ECSA* [31,32,33]. Therefore, we believe that the different *ECSA*_CO_ mainly originated from the effect of particle size and structure.

**Figure 5:**
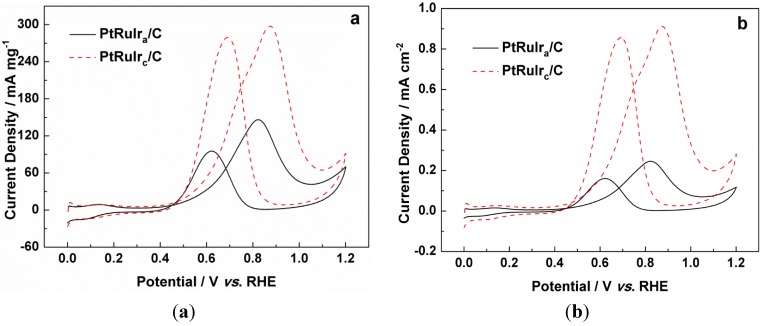
Cyclic voltammograms of PtRuIr_c_/C and PtRuIr_a_/C catalysts normalized to the metal loading on the electrodes (**a**) and *ECSA*_CO_ (**b**), in 0.5 mol L^−1^ CH_3_OH + 0.5 mol L^−1^ H_2_SO_4_ solution under N_2_ atmosphere; scan rate = 50 mV s^−1^.

The electrocatalytic activity of PtRuIr_c_/C and PtRuIr_a_/C catalysts in methanol oxidation is shown in Figure 5. The onset potential and the activity for methanol oxidation on both catalysts are shown in Table 1. In the forward scan in Figure 5a, the current density (mass activity) of PtRuIr_c_/C (298 mA mg^−1^) is 50% higher than that of PtRuIr_a_/C (147 mA mg^−1^). In Figure 5b, the current density (specific activity) of the PtRuIr_c_/C is 3.6 times as large as that of PtRuIr_a_/C. Although the particle size of PtRuIr_c_ is obviously larger than that of PtRuIr_a_, the PtRuIr_c_/C showed superior catalytic activity to PtRuIr_a_/C, *i.e.*, lower onset potential, and higher oxidation current density due to the effect of the structure. Moreover, the mass and specific activities of PtRuIr_c_/C are distinctly higher than those of the PtRuIr_a_/C catalyst (see Table 1).

**Figure 6:**
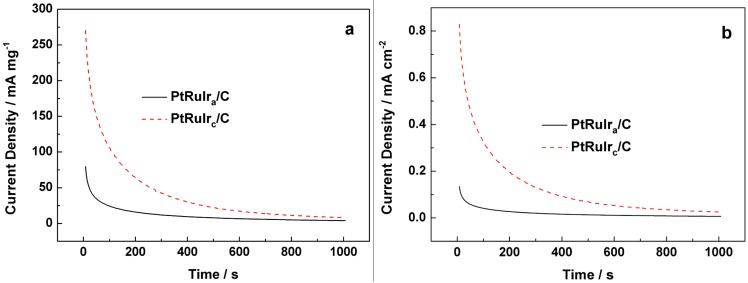
Chronoamper ometry curves of PtRuIr_a_/C and PtRuIr_c_/C catalysts for methanol oxidation, polarized at a constant potential of 0.6 V *vs*. Ag/AgCl at room temperature.

Figure 6 shows the chronoamperometry curves for the PtRuIr_c_/C and PtRuIr_a_/C catalysts in 0.5 mol L^−1^ H_2_SO_4_ and 0.5 mol L^−1^ CH_3_OH at a constant potential of 0.8 V (*vs*. RHE), the current density is normalized to the metal loading on the electrodes (a) and *ECSA*_CO_ (b), respectively. Figure 6, shows that the potentiostatic current for all the catalysts initially decreased rapidly owing to the formation of CO_ads_ and other intermediate species during the methanol oxidation reaction. With time, the current density decayed more gradually and a pseudo-steady state was achieved. The decay can be attributed to the adsorbed anion SO_4_^2−^ on the surface of the catalyst, thus restricting the methanol oxidation reaction. We calculated the long-term poisoning rate (*δ*) by measuring the linear decay of the current for a period of more than 500 s from Figure 6 by the following Equation (2) [33]:

(2) $$\delta =\frac{100}{i_{0}}\times {\left(\frac{di}{dt}\right)}_{t>500}$$

where ${\left(\frac{di}{dt}\right)}_{t>500}$ is the slope of the linear portion of the current decay and *i_0_* is the current at the start of polarization back extrapolated from the linear current decay. The current densities of the PtRuIr_c_/C at 1000 s are 8.30 mA mg^−1^ and 0.025 mA cm^−2^, while those of PtRuIr_a_/C catalysts at 1000 s are 4.0 mA mg^−1^ and 0.0067 mA cm^−2^, respectively. The calculated *δ* values show that the poisoning rate 0.10 of the PtRuIr_a_/C catalysts is slow compared to 0.13 of the PtRuIr_c_/C catalyst. This indicates that the PtRuIr_a_/C catalyst had a relatively lower poisoning rate than the PtRuIr_c_/C catalyst. Thus, although the PtRuIr_c_/C catalyst had a larger *ECSA*_CO_, the poisoning rate was faster than that of the PtRuIr_a_/C catalyst. This is probably because the faster and higher activities for the methanol oxidation reaction on the PtRuIr_c_/C electrode generated a larger amount of reactive intermediates and the ultimate poisoning species, rapidly producing the larger *δ* value.

## 3. Experimental Section

### 3.1. Preparation of PtRuIr/C Catalysts with Different Crystallinity

Amorphous PtRuIr/C catalyst (PtRuIr_a_/C) was prepared by a modified organic colloid method in ethylene glycol (EG) solution. In a typical process, a PtRuIr_a_/C catalyst with a nominal weight Pt:Ru:Ir ratio of 3:3:1 was prepared as follows: 4.85 mL 20 mg mL^−1^ H_2_PtCl_6_·aqueous solutions, 1.99 mL 20 mg mL^−1^ RuCl_3_, 2.56 mL 10 mg mL^−1^ H_2_IrCl_6_ and sodium citrate (220 mg) were dissolved in 30 mL ethylene glycol (EG) and stirred for 0.5 h. Pretreated carbon black Vulcan^®^ XC72R (100 mg) was added to the mixture under stirring conditions. The pH of the system was adjusted to ~9 by drop-wise addition of a 5 wt % KOH/EG solution with vigorous stirring. The mixture was transferred to a flask and heated at 160 °C for 6 h and the resultant product was collected by filtration, washed with ultrapure water to remove all residual chloride ion, and then dried in air at 60 °C for 12 h. The metal loading is 20%. Crystalline PtRuIr/C catalyst (PtRuIr_c_/C) was prepared by heating the above-prepared PtRuIr_a_/C powder in a tube furnace under H_2_/N_2_ atmosphere at 700 °C for 2 h.

### 3.2. Measurements

The catalysts were characterized by recording their XRD patterns on a Shimadzu XD–3A (Japan), using filtered Cu-Kα radiation (*λ* = 0.15418 nm), generated at 40 kV and 30 mA. Scans for 2*θ* values were recorded at 4°/min between 10° and 90°. All X–ray diffraction patterns were analyzed using Jade 7.5 of Material Data, Inc. (MDI): peak profiles of individual reflections were obtained by a nonlinear least-square fit of the Cu-Kα corrected data. TEM measurements were carried out on a Tecnai G220 S–TWIN (FEI Company); the acceleration voltage was 200 kV. The average chemical compositions of the two catalysts were determined by the energy-dispersive X-ray spectroscopy (EDS) analysis and IRIS advantage inductively coupled plasma atomic emission spectroscopy (ICP-AES) system (Thermo Electron Corporation, America).

A common three-electrode cell was used for the electrochemical measurements, using a CHI 650D electrochemical work station. The counter and reference electrode were a platinum wire and an Ag/AgCl (3 M KCl) electrode, respectively, and the working electrode was a glassy carbon disk (5 mm in diameter). The thin-film electrode was prepared as follows: 5 mg of catalyst was dispersed ultrasonically in 1 mL Nafion/ethanol (0.25% Nafion) for 15 min. 8 μL of the dispersion was transferred onto the glassy carbon disk using a pipette, and then dried in the air. The metal loading on the film is 40.8 μg cm^−2^.

## 4. Conclusions

Carbon-supported PtRuIr alloy catalysts of amorphous and crystalline structure were successfully synthesized and characterized. Electrochemical characterization found that although PtRuIr_a_/C had a larger electrochemical surface area mainly due to the small size of the particles, the PtRuIr_c_/C had the better electrochemical performance in the methanol oxidation reaction. However, the poisoning rate of the PtRuIr_c_/C catalyst was faster than that on the PtRuIr_a_/C catalyst. The difference in activity originates from the effect of the structure. Therefore, these results show that control of crystallinity of the active alloy nanoparticles can play an important role in both methanol oxidation activity and in the CO poisoning rate.
